# ﻿Two new species of freshwater planarian from Hainan Island and Leizhou Peninsula, southern China (Platyhelminthes, Tricladida, Dugesiidae)

**DOI:** 10.3897/zookeys.1233.142976

**Published:** 2025-04-01

**Authors:** Lei Wang, Yi-Fang Chang, Xin-Xin Sun, Ronald Sluys, De-Zeng Liu, Zi-Mei Dong, Guang-Wen Chen

**Affiliations:** 1 College of Life Science, Henan Normal University, Xinxiang, 453007 Henan, China Henan Normal University Xinxiang China; 2 Naturalis Biodiversity Center, Leiden, Netherlands Naturalis Biodiversity Center Leiden Netherlands

**Keywords:** *Dugesia*, new species, southern China, taxonomy, triclads

## Abstract

Two new species of the genus *Dugesia* from Hainan Island and Leizhou Peninsula are described by applying an integrative approach, including morphological, karyological, histological, and molecular information. In the molecular phylogenetic tree, the two new species, *Dugesiasaccata* Chen & Dong, **sp. nov.** and *Dugesiaaconcinna* Chen & Dong, **sp. nov.**, fall into an Eastern Palearctic/Oriental clade and an Oriental/Australasian clade, respectively, while sharing only a rather distant relationship. The separate specific status of the two new species is supported also by their genetic distances. *Dugesiasaccata* is characterised by the presence of the following features: a sac-shaped expansion at the knee-shaped bend of the bursal canal; ventrally displaced ejaculatory duct with a subterminal opening; a duct between diaphragm and seminal vesicle; mixoploid karyotype with diploid complements of 2n = 2x = 16 and triploid complements of 2n = 3x = 24, with all chromosomes being metacentric. *Dugesiaaconcinna* is characterised by the presence of the following features: asymmetrical openings of the oviducts into the bursal canal and the common atrium, with the left oviduct opening into the common atrium and the right oviduct opening into the most ventral, proximal portion of the bursal canal, at the point where the latter communicates with the common atrium; vasa deferentia separately opening into the posterior portion of the seminal vesicle; penis papilla of a very characteristic shape, with the part housing the connecting duct, diaphragm, and ejaculatory duct being a cylindrical structure with a blunt tip, while at its right-hand side sits a large penial fold that attaches to the base of the penis papilla; ejaculatory duct following a ventrally displaced course through the penis papilla, after which it opens at the tip of the papilla; presence of a duct between diaphragm and seminal vesicle.

## ﻿Introduction

To date, approximately 110 species of freshwater planarians of the genus *Dugesia* Girard, 1850 have been reported from the Afrotropical, Palearctic, Oriental and Australasian biogeographic regions (Dols-Serrate et al. 2024). Over the past five years, 14 species of *Dugesia* have been described from China, from which ten species occur in Southern China (Chen et al. 2022; Wang et al. 2024; Zeng et al. 2024; Zhu et al. 2024, and references therein). These recent taxonomic studies revealed a rich biodiversity in southern China, which forms a potential distribution hotspot for *Dugesia* (Solà et al. 2022; Wang et al. 2024).

**Figure 1.**
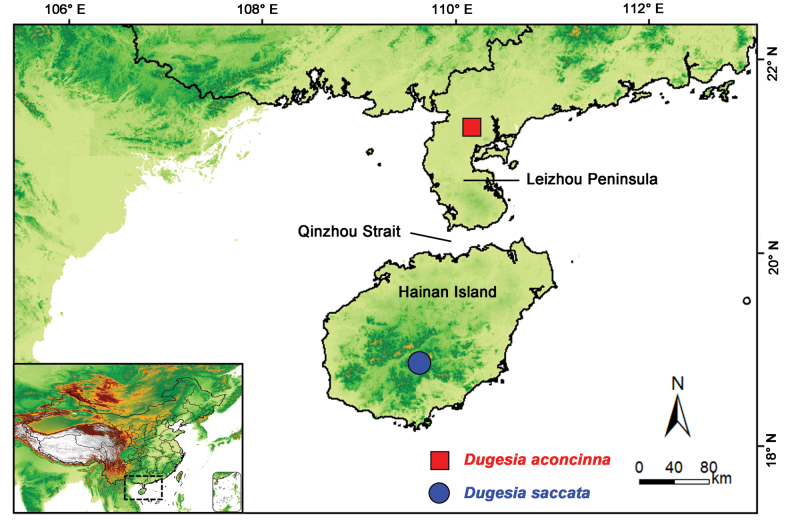
Collection sites of *Dugesia* in Hainan Island and Leizhou Peninsula.

Hainan Island is a tropical island and the second largest island in southern China from which many species of insects have been reported (Li et al. 2020, 2024). The island lies opposite to the Leizhou Peninsula, the third largest peninsula in China, separated from it by the Qinzhou Strait, which forms a natural geographic barrier between the island and the peninsula (Fig. 1). Its special physical geographic environment contributes greatly to the fact that Hainan Island forms one of China’s hotspots of biodiversity (Bu et al. 2019). The species diversity on Hainan Island includes the freshwater planarians *Dugesiamajuscula* Chen & Dong, 2021 and *D.semiglobosa* Chen & Dong, 2021. In the present study we describe a third new species of *Dugesia* for Hainan Island as well as the first and equally new species of *Dugesia* for the Leizhou Peninsula, by applying an integrative approach, including morphological, karyological, histological, and molecular information, albeit the chromosomes could be examined for only one of the new species.

## ﻿Materials and methods

### ﻿Specimen collection and culturing

Specimens were collected from under stones in streams or springs with the help of a paintbrush. After collection, the worms were transferred to plastic bottles filled with stream water that during transportation to the laboratory were placed in a cooler filled with an ice bag. In an automatic incubator (BOXUN BSP-800) the planarians were cultured in autoclaved tap water at 20 °C and fed with fresh beef liver once per week. The worms were starved for at least seven days before being used for karyotype and histological studies and DNA extraction. Images of their external morphology were obtained by using a digital camera attached to a stereo-dissecting-microscope (Leica M165C).

In order to distinguish between sympatric populations at the same collection site, all worms were at first carefully examined in the laboratory under the stereo-microscope and separated into groups based on morphological differen­ces. Hereafter, randomly selected individuals from each group or population (at least 3–5 worms from each population, including sexual and asexual individuals), were cut into two fragments. The anterior fragments were used for DNA extraction, amplification, and sequencing; the posterior fragments were used for histological or karyological studies. Sexual individuals from each population were used to prepare histological sections, irrespective whether they had been collected sexually mature in the field or had sexualised in the laboratory.

### ﻿Phylogenetic analysis and genetic distances

Procedures for DNA extraction, amplification and sequencing followed Wang et al. (2021a). The quality and quantity of DNA was determined by NanoDrop oneC (Thermo Scientific). For both new species, four specimens were used to extract DNA, and four gene fragments were amplified by polymerase chain reaction (PCR), namely 18S ribosomal gene (*18S rDNA*, type II), 28S ribosomal gene (*28S rDNA*), ribosomal internal transcribed spacer-1 (*ITS-1*), and Cytochrome C oxidase subunit I (*COI*). Primers used for amplification and the PCR protocol are listed in Suppl. material 1. In total, we generated datasets consisting of four gene sequences (*18S rDNA*, *28S rDNA*, *ITS-1*, and *COI*) of the two new *Dugesia* species and available sequences of other *Dugesia* species from major portions of the geographic range of the genus, while *Schmidteamediterranea* (Benazzi et al., 1975), *S.polychroa* (Schmidt, 1861), and *Recurvapostrema* Sluys & Solà, 2013, were chosen as the outgroup taxa to perform phylogenetic analyses (Table 1).

**Table 1.** GenBank accession numbers of sequences used in molecular analyses. New species indicated in boldface.

Species	*COI*	*ITS-1*	*28S*	*18S*
***D.aconcinna***	PV055688	PV055833	PV055834	–
*D.adunca*	OL505739	OL527659	–	–
*D.aethiopica*	KY498845	KY498785	KY498806	KY498822
*D.afromontana*	KY498846	KY498786	KY498807	KY498823
*D.ancoraria*	OR326966	OR296750	OR225689	OR198141
*D.arabica*	OL410620	OK587374	OK491342	OK646637
*D.arcadia*	KC006971	KC007044	OK491318	KF308694
*D.ariadnae*	KC006972	KC007048	OK491317	OK646636
*D.batuensis*	OL410626	OK587362	OK491316	OK646630
*D.benazzii*	FJ646977 + FJ646933	FJ646890	MK712509	OK646628
*D.bengalensis*	–	FJ646897	–	–
*D.bifida*	KY498851	KY498791	KY498813	KY498843
*D.bijuga*	MH119630	–	–	MH113806
*D.circumcisa*	MZ147041	MZ146782	–	–
*D.cretica*	KC006976	KC007050	OK491340	KF308697
*D.constrictiva*	MZ871766	MZ869023	–	–
*D.damoae*	KF308768	KC007057	OK491310	OK646619
*D.deharvengi*	KF907820	KF907817	KF907824	–
*D.effusa*	KF308780	KC007058	OK491311	OK646618
*D.elegans*	KC006984	KC007063	OK491313	KF308695
*D.etrusca*	FJ646984 + FJ646939	FJ646898	OK491312	OK646617
*D.gemmulata*	OL632201	–	–	–
*D.gibberosa*	KY498857	KY498803	KY498819	KY498842
*D.gonocephala*	FJ646986 + FJ646941	FJ646901	DQ665965	DQ666002
*D.granosa*	OL410634	KY498795	KY498816	KY498833
*D.hepta*	MK712639	MK713035	OK491309	OK646612
*D.hoidi*	OR650791	–	–	–
*D.improvisa*	KC006987	KC007065	OK491304	KF308696
*D.japonica*	FJ646990	FJ646904	DQ665966	D83382
*D.liguriensis*	OL410632	OK587358	OK491353	OK646615
*D.malickyi*	KC006988	KC007068	OK491294	OK646585
*D.majuscula*	MW533425	MW533591	–	–
*D.mariae*	OR650829	–	–	–
*D.musculosa*	OR189184	OR205922	–	–
*D.naiadis*	KF308756	OK587343	OK491293	–
*D.notogaea*	FJ646993 + FJ646945	FJ646908	KJ599720	KJ599713
*D.parasagitta*	KF308739	KC007073	–	OK646577
*D.pendula*	OR195337	OR205921	–	–
*D.pustulata*	MH119631	OK587366	OK491355	MH113807
*D.ryukyuensis*	AB618488	FJ646910	OK491323	AF050433
***D.saccata***	PV055687	PV055830	PV055832	PV055831
*D.sagitta*	KC007006	KC007077	OK491320	OK646567
*D.semiglobosa*	MW525210	MW526992	–	–
*D.sicula*	KF308797	OK587339	OK491287	KF308693
*D.sigmoides*	KY498849	KY498789	KY498811	KY498827
*D.sinensis*	KP401592	–	–	–
*D.subtentaculata*	MK712628	MK713004	MK712501	AF013155
*D.tumida*	OL505740	OL527709	–	–
*D.umbonata*	MT176641	MT177211	MT177210	MT177214
*D.verrucula*	MZ147040	MZ146760	–	–
*R.postrema*	KF308763	–	MG457274	KF308691
*S.mediterranea*	JF837062	AF047854	DQ665992	U31085
*S.polychroa*	FJ646975 + FJ647021	–	DQ665993	AF013152

Sequence analyses were done as described previously by Wang et al. (2024). In brief, *18S rDNA*, *28S rDNA* and *ITS-1* sequences were aligned online with MAFFT (Online version 7.247) using the G-INS-i algorithm. Protein-coding sequences (i.e., *COI*) were translated into amino acid sequences in order to check for the presence of stop codons (with the NCBI’s genetic code 9 – Flatworm Mitochondrial). For *COI*, sequences were aligned online with Translator X (Abascal et al. 2010, http://translatorx.co.uk) using FFT-NS-2 method, were checked by BioEdit 7.2.6.1 (Hall 1999) and, thereafter, back-translated to nucleotide sequences. Since automated removal of gap columns and variable regions has been reported to negatively affect the accuracy of the inferred phylogeny (Dessimoz and Gil 2010; Tan et al. 2015), the Gblocks option was disabled (Talavera and Castresana 2007). A total of three datasets were used in this study, viz., dataset I: *ITS-1* (46 sequences; Table 1), dataset II: *COI* (52 sequences; Table 1), dataset III: concatenated sequences *COI* + *ITS-1* + *28S rDNA + 18S rDNA* (171 sequences; Table 1). The substitution saturation test for *COI* (using DAMBE 6, according to Xia et al. 2003) showed no sign of saturation.

Bayesian information criterion (BIC) was implemented in PartitionFinder 2 (Lanfear et al. 2017) to estimate the best-ﬁt partition schemes and models of dataset III. The best models for each gene and codon position were as follows: *ITS-1*: GTR + G;*18S rDNA* and *28S rDNA*: GTR + I + G; *COI*: GTR + I + G for the first and third codon positions, and HKY + I + G for the second codon position. Bayesian inference analysis (BI) was run with MrBayes v. 3.2 (Ronquist et al. 2012) using two replicate runs with four chains for 5 million generations, sampling trees every 1000 generations. The convergence of runs was checked by monitoring that the standard deviation of split frequencies reached a value below 0.01, thus indicating that the runs had reached a stationary state. Following completion of each analysis, the ﬁrst 25% of the generated trees were discarded as “burn-in”. Maximum likelihood (ML) analysis with IQ-TREE (Minh et al. 2020) was used to perform 10,000 replicates of ultrafast bootstrap approximation (Hoang et al. 2018). BI and ML trees were visualised and edited using Figtree v. 1.4.3. Genetic distan­ces, based on dataset I and dataset II, were calculated by MEGA 6.06 (Tamura et al. 2013) with the Kimura 2-parameter substitution model (Solà et al. 2013).

### ﻿Histology and karyology

Histological sections were prepared as described previously by Dong et al. (2017). In brief, worms were fixed in Bouin’s fluid for 24 h, and, subsequently, rinsed and stored in 70% ethanol. For histological study, specimens were dehydrated in an ascending series of ethanol solutions, after which they were cleared in clove oil and embedded in synthetic wax. Serial sections were made at intervals of 6 μm and were stained with Hematoxylin-Eosin (cf. Winsor and Sluys 2018). Histological preparations of specimens have been deposited in the Zoological Museum of the College of Life Science of Henan Normal University, Xinxiang, China (**ZMHNU**), and Naturalis Biodiversity Center, Leiden, The Netherlands (**RMNH**).

The air-drying method was used to obtain karyological preparations (Wang et al. 2024), while the karyological analysis was done with a compound microscope (ZEISS, Axio Scope. A1) equipped with CoolCube digital camera (MetaSystems, Altlussheim, Germany). Karyograms were prepared using the IKAROS Karyotyping system (MetaSystems, Altlussheim, Germany, https://metasystems-international.com/en/products/ikaros/), according to the protocols described by Wang et al. (2024). For details on preparation and analyses of karyology, see Extended methods in Suppl. material 2.

## ﻿Results

### ﻿Molecular phylogeny and genetic distances

Phylogenetic trees were constructed using the alignment of 4308 base pairs (bp), including 1564 bp for *18S rDNA*, 1383 bp for *28S rDNA*, 656 bp for *ITS-1*, and 705 bp for *COI* (Table 1). Four specimens were examined from each of the two new species, *Dugesiasaccata* and *D.aconcinna*, and these showed no variation in either of the four gene sequences.

**Figure 2.**
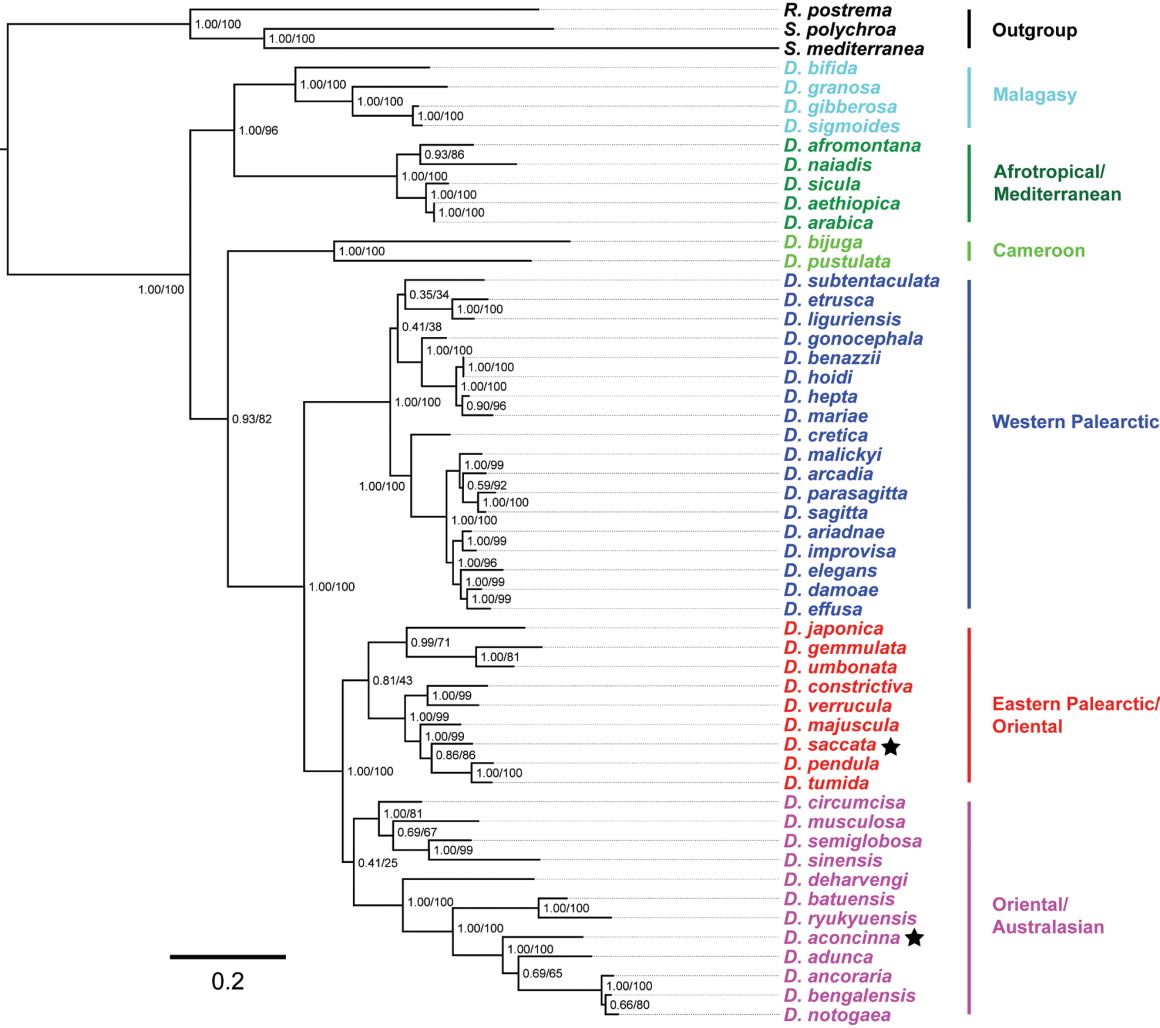
Topology of the molecular phylogenetic tree obtained from Bayesian analysis of the concatenated dataset (dataset III). Numbers at nodes indicate support values (pp/bs). New species indicated by asterisks. Scale bar: substitutions per site.

The BI and ML generated trees showed identical topologies, differing only in support values (Fig. 2, Suppl. material 3). The two new species fall into two major groups, viz., an Eastern Palearctic/Oriental group and an Oriental/Australasian group, albeit these are statistically poorly supported and, thus effectively form one polytomous group of species. Nevertheless, within this group the clade of the two species *D.pendula* Chen & Dong, 2024 and *D.tumida* Chen & Sluys, 2022 shares a sister-group relationship with *D.saccata*, with rather good support (pp = 86, bs = 0.86). The four species *D.adunca*, *D.ancoraria* Zhu & Wang, 2024, *D.bengalensis* Kawakatsu, 1983, *D.notogaea* Sluys & Kawakatsu, 1998 and *D.aconcinna* clearly cluster together, forming a clade with high support (pp = 100, bs = 1.00).

The highest *COI* distance values between *D.saccata* and *D.aconcinna* and their congeners were 21.54% (with *D.bijuga* Harrath & Sluys, 2019) and 22.75% (with *D.naiadis* Sluys, 2013), respectively, while the lowest *COI* distance values were 8.78% and 10.87% (both with *D.deharvengi* Kawakatsu & Mitchell, 1989). Furthermore, there is a 18.48% *COI* difference between the two new species (Suppl. material 4). With respect to *ITS-1*, *D.saccata* and *D.aconcinna* showed the highest distance values with *D.pustulata* Harrath & Sluys, 2019 and *D.naiadis* Sluys, 2013, being 19.60% and 21.25%, respectively, and exhibited the lowest distance values with *D.pendula* (1.76%) and *D.adunca* (2.97%). For *ITS-1*, the molecular distance between the two new species was 10.85% (Suppl. material 5). Thus, the separate species status of *D.saccata* and *D.aconcinna* is well-supported by both molecular phylogenetics and genetic distances.

### ﻿Systematic account

#### ﻿Order Tricladida Lang, 1884

**Suborder Continenticola Carranza, Littlewood, Clough, Ruiz-Trillo, Baguñà & Riutort, 1998**

**Family Dugesiidae Ball, 1974**

**Genus *Dugesia* Girard, 1850**

##### ﻿ Dugesia saccata

Taxon classificationAnimaliaTricladidaDugesiidae

Chen & Dong sp. nov.

6A0099F7-0FD4-5834-BE0B-3762F9816AF2

https://zoobank.org/079234B5-51CF-4876-AFCD-C83ADD28B46A

**Figure 3.**
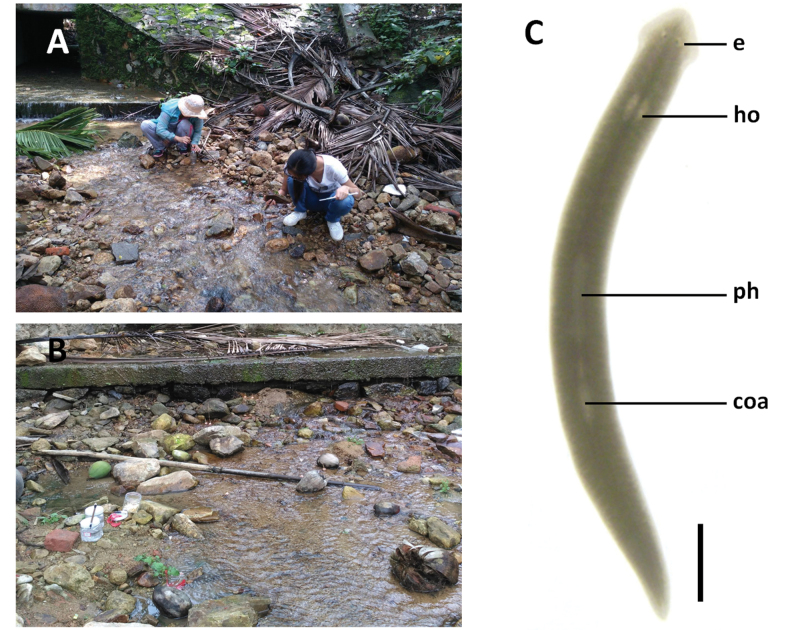
Habitat and external appearance of *Dugesiasaccata***A, B** sampling site and habitat **C** sexually mature, live individual. Abbreviations: coa: copulatory apparatus; e: eye; ho: hyperplasic ovary; ph: pharynx. Scale bar: 2 mm.

**Figure 4.**
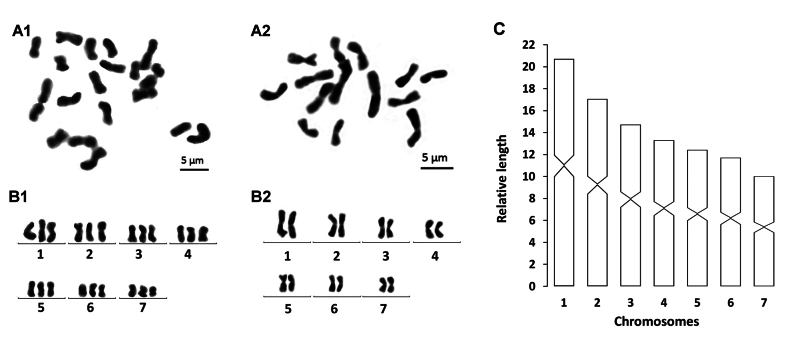
*Dugesiasaccata***A1, B1** metaphase plate and karyogram of triploid complement **A2, B2** metaphase plate and karyogram of diploid complement **C** idiogram. Scale bars: 5 μm.

**Figure 5.**
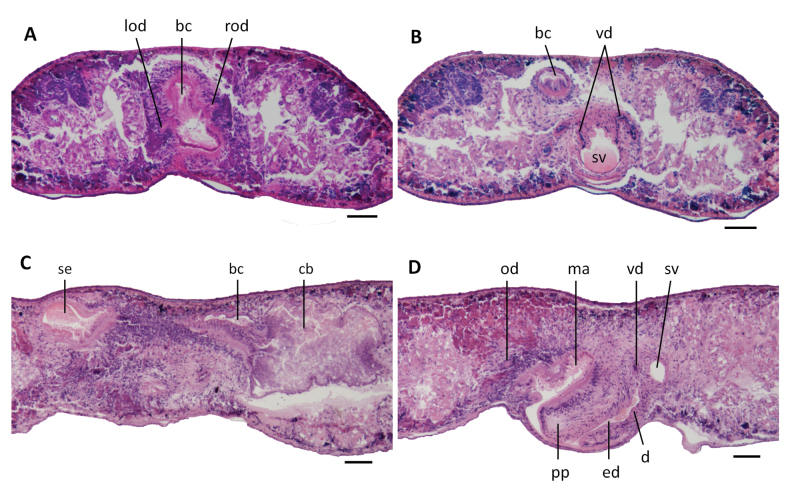
*Dugesiasaccata*. Photomicrographs of transverse (**A, B**) and sagittal (**C, D**) sections. **A** Paratype ZMHNU-YZCB8, showing symmetrical openings of oviducts into the bursal canal **B** paratype ZMHNU-YZCB8, showing symmetrical openings of vasa deferentia into the seminal vesicle **C** holotype ZMHNU-YZCB1, showing copulatory bursa and bursal canal **D** holotype ZMHNU-YZCB1, showing penis papilla, vasa deferentia, seminal vesicle, diaphragm, and ejaculatory duct. Abbreviations: bc: bursal canal; cb: copulatory bursa; d: diaphragm; ed: ejaculatory duct; lod: left oviduct; ma: male atrium; od: oviduct; pp: penis papilla; rod: right oviduct; se, sac-shaped expansion of bursal canal; sv: seminal vesicle; vd: vas deferens. Scale bars: 100 μm.

**Figure 6.**
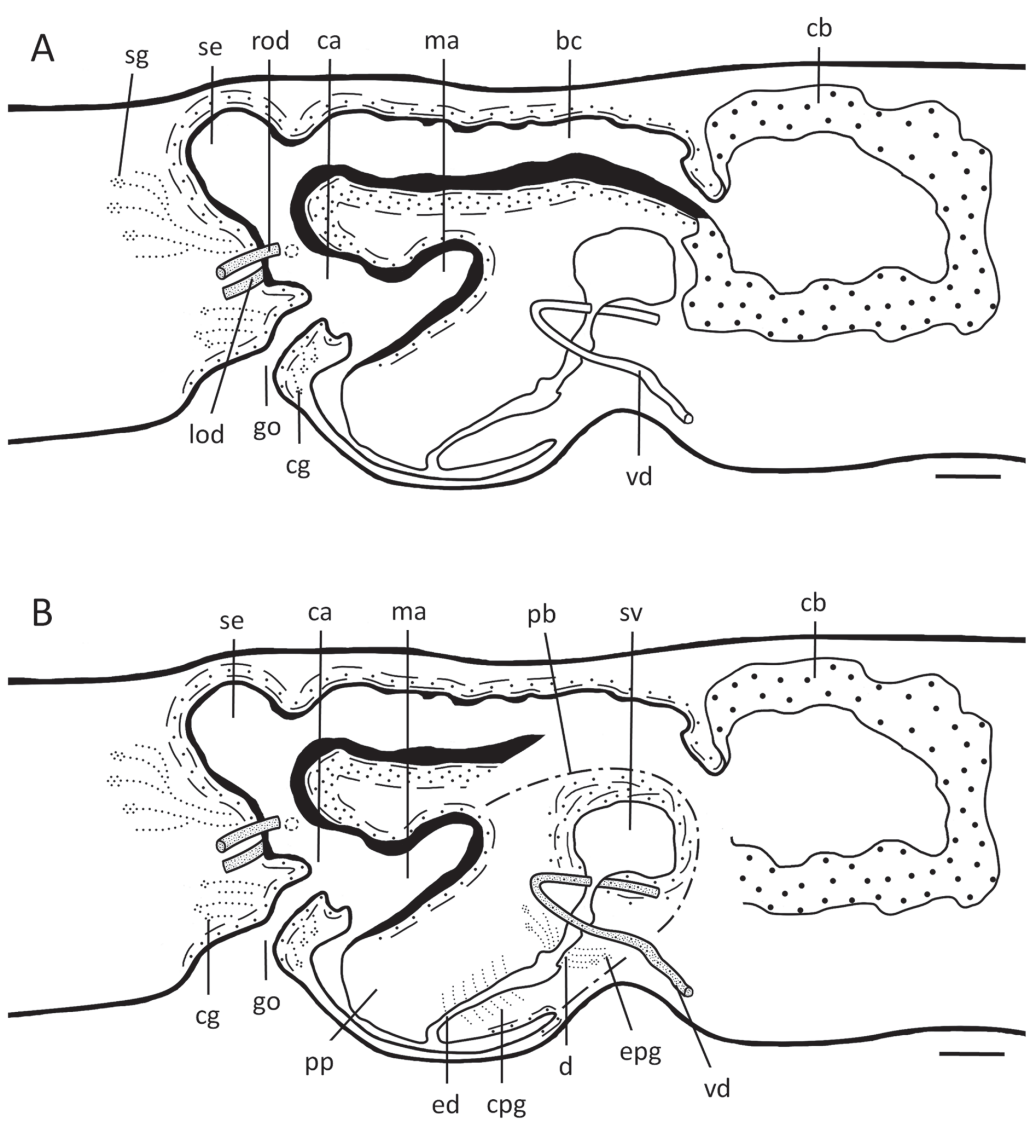
*Dugesiasaccata*. Sagittal reconstruction of the copulatory apparatus of holotype YZCB1 **A** female copulatory apparatus **B** male copulatory apparatus. Abbreviations: bc: bursal canal; ca: common atrium; cb: copulatory bursa; cg: cement glands; cpg: cyanophil penial glands; d: diaphragm; ed: ejaculatory duct; epg: extrabulbar penial glands; go: gonopore; lod: left oviduct; ma: male atrium; mo: mouth; pb: penis bulb; pp: penis papilla; rod: right oviduct; se, sac-shaped expansion of bursal canal; spv: spermiducal vesicles; sg: shell glands; sv: seminal vesicle; vd: vas deferens. Scale bars: 100 μm.

Figs 1 , 2 , 3 , 4 , 5 , 6

###### Collection site, habitat, and reproduction.

On 24 February 2018, specimens were collected from a freshwater stream in the Yingge Mountains, Hainan Island (Figs 1, 3A), which is located within a national Nature Reserve at an altitude of 430 m a.s.l.; air temperature was 24 °C and water temperature was 21 °C . In the population of *D.saccata*, all worms were asexual at collection in the field, and under laboratory conditions were fissiparous. During a period of ~ 4 months, each of the ten specimens sexualised; in the first month only two sexualised individuals were found, while at the end of the fourth month ten sexual animals were present. After 18 months of culturing, sexualised worms produced > 10 cocoons. The spherical cocoons (1.3 mm in diameter) were dark brownish and provided with a stalk. Thus far, none of the cocoons hatched, most likely infertile. During laboratory culturing, the sexualised worms sometimes lost their copulatory apparatus and, subsequently, returned to the asexual mode of reproduction.

###### Material examined.

***Holotype*** • ZMHNU-YZCB1, Yongzhong village (18°46'6"N, 109°38'42"E), alt. 430 m a.s.l., Wuzhishan City, Hainan Province, China, 24 February 2018, coll. G-W Chen, D-Z Dong and co-workers, sagittal sections on 28 slides. ***Paratypes*** • RMNH.VER.22249.1, ibid., sagittal sections on 12 slides • RMNH.VER.22249.2, ibid., sagittal sections on 20 slides • ZMHNU-YZCB2, 3, 6, ibid., sagittal sections on 19, 18, and 26 slides, respectively • ZMHNU-YZCB5, ibid., horizontal sections on 16 slides • ZMHNU-YZCB8, ibid., transverse sections on 35 slides.

###### Diagnosis.

*Dugesiasaccata* is characterised by the presence of the following features: symmetrical openings of the oviducts into the most proximal section of the bursal canal, near the point where the latter communicates with the atrium; a sac-shaped expansion at the knee-shaped bend of bursal canal; vasa deferentia opening symmetrically into posterior portion of the seminal vesicle; ventrally displaced ejaculatory duct with subterminal opening; a duct between diaphragm and seminal vesicle; mixoploid karyotype, with diploid chromosome portraits of 2n = 2x = 14, and triploid complements of 2n = 3x = 21, with all chromosomes being metacentric.

###### Karyology.

Seven intact specimens were randomly selected to prepare metaphase plates. In a total of 157 metaphase plates that were examined, 42 plates exhibited diploid chromosome complements of 2n = 2x = 14, while in 104 plates chromosome complements were triploid with 2n = 3x = 21 chromosomes; chromosome complements on the remaining 11 plates could not be determined, due to either lack of well dispersed chromosomes or over-dispersed sets of chromosomes. All seven specimens exhibited mixoploid chromosome complements, with all chromosomes being metacentric. Karyotype parameters, including relative length, arm ratio, and centromeric index, are given in Table 2. Chromosomal plates and an idiogram are shown in Fig. 4.

**Table 2.** Karyotype parameters (mean values and standard deviations) of *Dugesiasaccata*.

Chromosome	Relative length	Arm ratio	Centromeric index	Chromosome type
1	19.00 ± 0.41	1.09 ± 0.05	47.91 ± 1.08	metacentric
2	16.74 ± 0.46	1.24 ± 0.12	44.85 ± 2.44	metacentric
3	15.03 ± 0.25	1.15 ± 0.05	46.69 ± 0.98	metacentric
4	14.00 ± 0.31	1.14 ± 0.07	46.93 ± 1.52	metacentric
5	13.08 ± 0.43	1.09 ± 0.03	48.00 ± 0.65	metacentric
6	12.00 ± 0.11	1.14 ± 0.05	46.78 ± 1.06	metacentric
7	10.26 ± 0.61	1.15 ± 0.03	46.74 ± 0.68	metacentric

###### Morphology.

In sexualised living specimens, the body measured 14–22 mm in length and 1.3–1.6 mm in width. Triangular head provided with two blunt auricles and two eyes, which are placed in pigment-free spots. Each pigmented eyecup houses numerous photoreceptor cells. The dorsal surface is taupe, the ventral surface is paler in colour than the dorsal one (Fig. 3C).

Pharynx situated at the mid-region of the body, measuring ~ 1/5 of the body length. Mouth opening located at posterior end of the pharyngeal pocket. The outer pharyngeal musculature is composed of a subepithelial layer of longitudinal muscles, followed by a layer of circular muscles. The inner pharyngeal musculature consists of a thick subepithelial layer of circular muscles, followed by a thin layer of longitudinal muscles.

The hyperplasic ovaries are located at 1/3–1/5 of the distance between the brain and the root of the pharynx, occupying ~ 1/2 of the dorso-ventral space, with several scattered masses. The oviducts arise from the dorsal wall of the ovaries, then turn to the ventral side and run in a caudal direction to the level of the genital pore, after which they curve dorso-medially to open separately and symmetrically into the bursal canal, near the point where the latter communicates with the atrium (Fig. 5A). Cyanophil shell glands discharge their secretion into the vaginal region of the bursal canal, at the level of the oviducal openings.

The small, dorsally located testes are poorly developed and provided with only a few mature spermatozoa. As a consequence, we found spermatozoa to be present in the vasa deferentia only in specimens YZCB3, 5, 6, and 8, as well as in RMNH.VER.22249.2. Testicular follicles are arranged on either side of the midline of the body in four or five longitudinal zones, extending from the posterior level of the ovaries to almost the posterior end of the body.

At the level of the penis bulb, the sperm ducts curve towards the dorsal body surface, then penetrate the ventral wall of the penis bulb to open separately into the seminal vesicle. The precise approach of the ducts to the seminal vesicle differs somewhat between specimens. In the holotype one sperm duct exhibits a short dorso-ventral recurvature before opening into the proximal section of the duct that leads from the seminal vesicle to the diaphragm; the other duct opens at the same position but has a much more direct approach (Fig. 6). In paratype YZCB-6 there is an even more distinctly asymmetrical approach of the sperm ducts, with one duct having a dorsal approach, after a well-developed dorso-ventral recurvature, opening in the antero-lateral portion of the seminal vesicle, again close to the duct leading to the diaphragm. The other sperm duct does not show the recurvature and opens directly into the proximal section of the connecting duct between seminal vesicle and diaphragm. In contrast, in paratype YZCB-8 both ducts exhibit a well-developed recurvature before symmetrically opening into the latero-dorsal portions of the seminal vesicle, close to the point where the connecting duct opens into the seminal vesicle (Fig. 5B).

The sperm ducts are lined with nucleated cells and surrounded by a layer of circular muscles. The oval-shaped, rather large seminal vesicle is lined by a flat, nucleated epithelium and is surrounded by a layer of irregularly crosswise arranged muscle fibres. The postero-ventral section of the seminal vesicle gives rise to a duct that is lined by an infranucleated epithelium, which is underlain by a subepithelial layer of intermingled muscle fibres and via a small diaphragm opens into the ejaculatory duct (Figs 5D, 6). The small diaphragm is located at approximately the root of the penis papilla and receives the abundant secretion of erythrophil penis glands (Fig. 5D). The ejaculatory duct, which is lined with a cuboidal, infranucleated epithelium, is devoid of any discernible musculature and follows a ventrally displaced course through the penis papilla, opening subterminally at its tip (Figs 5D, 6). Cyanophil penis glands discharge abundant secretion into the central and distal portion of the ejaculatory duct.

Because of the ventrally displaced course of the ejaculatory duct, the penis papilla is asymmetrical, with its dorsal lip being considerably larger than the ventral one (Figs 5D, 6). The cylindrical penis papilla has an oblique, ventro-caudal orientation and is covered by a nucleated epithelium, which is underlain by a subepithelial layer of circular muscle, followed by a layer of longitudinal muscle fibres (Figs 5D, 6).

The copulatory bursa is a large sac-shaped structure that may occupy the entire dorso-ventral space (paratype YZCB-6), while in other specimens it extends well over the central longitudinal axis of the body but does not fully occupy the dorso-ventral space (e.g., holotype YZCB-1; Fig. 6). The bursa is lined by a vacuolated epithelium with basal nuclei and is almost devoid of any surrounding musculature. The bursal canal arises from the postero-dorsal wall of the bursa, after which it runs in a caudal direction to the left side of the male copulatory apparatus (Figs 5C, 6A). The rather broad bursal canal occupies ~ 1/4 of the dorso-ventral space. At the level of the gonopore it decreases somewhat in diameter, whereafter it gives rise to a saccate posterior extension (Figs 5C, 6). The antero-ventral, knee-shaped bend in the bursal canal arises from the ventral wall of the saccate portion and communicates with the common atrium (Figs 5C, 6).

The bursal canal is lined with a ciliated epithelium with basal nuclei. Particularly the dorsal wall of the canal may be thrown into several folds. It is noteworthy that the ventral wall of the bursal canal is lined with cylindrical cells, whereas the dorsal wall is composed of cuboidal or even flat cells; the saccate expansion is also lined with a low epithelium. The bursal canal is surrounded by a subepithelial layer of longitudinal muscles, followed by a layer of circular muscle that is particularly well developed on the ventral wall of the canal; an extra outer layer of longitudinal musculature, forming the ectal reinforcement, extends from the atrium to 2/3 on the bursal canal.

The common atrium communicates with a gonoduct, which is lined by a columnar epithelium and receives the openings of erythrophil cement glands (Fig. 6).

###### Etymology.

The specific epithet is derived from the Latin noun *saccus*, bag, and alludes to the sac-shaped expansion at the knee-shaped bend of the bursal canal.

###### Discussion.

There is one character that immediately sets *D.saccata* apart from all of its known congeners, the sac-shaped expansion on the posterior section of the bursal canal, near the knee-shaped bend of the canal. This is slightly reminiscent of a situation in *Dugesiaaethiopica* Stocchino, Corso, Manconi & Pala, 2002, in which the posterior section of the bursal canal, immediately before receiving the separate openings of the oviducts, is expanded in lateral direction and gives rise to several large folds (Sluys 2007). However, this is merely a superficial resemblance to the situation in *D.saccata*, and these two species also differ in many other features. For example, *D.aethiopica* shows a horizontal approach of the bursal canal to the atrium, which represents a rare feature among species of *Dugesia* and is also absent in *D.saccata*. In *Dugesiaarabica* Harrath & Sluys, 2013 the bursal canal is considerably expanded as well as highly folded in the region of the oviducal openings (Harrath et al. 2013). However, this situation differs from that in *D.saccata* in that the expansion sits near the oviducal openings, whereas in *D.saccata* the sac-shaped expansion occurs dorsally, or entally to the openings of the oviducts into the bursal canal. Another difference between these two species concerns the presence of a duct between seminal vesicle and diaphragm in *D.saccata* and absence of such a duct in *D.arabica*.

Two characteristic features of *D.saccata* may be found also in other species of *Dugesia*, a ventrally displaced ejaculatory duct with subterminal opening and the presence of a duct between the seminal vesicle and the diaphragm. Besides *D.saccata*, these two character states are also expressed, among others, in the three Chinese species *D.majuscula*, *D.umbonata* Song & Wang, 2020, and *D.verrucula* Chen & Dong, 2021 (Song et al. 2020; Wang et al. 2021a, b). However, in contrast to *D.saccata*, in both *D.majuscula* and *D.umbonata* the ejaculatory duct has a subterminal dorsal opening at the tip of the penis papilla, while *D.verrucula* exhibits a permanent dorsal bump near the root of the penis papilla, which is absent in *D.saccata*. Another difference concerns the presence of a large muscularised hump on the dorsal surface of the bursal canal of *D.umbonata*, which is absent in *D.saccata*. Although these three species (*D.umbonata*, *D.verrucula*, and *D.saccata*) belong to the same clade, they are molecularly well-differentiated. On the other hand, while *D.saccata*, *D.pendula*, *D.tumida*, and *D.majuscula* belong to the same small clade, they are anatomically well differentiated.

In *Dugesia* species, the basic chromosome number concerns three types, 7, 8, and 9. Previous studies have shown that in China number 8 is the most frequent type, while 7 is much rarer (Wang et al. 2024). In that respect, it is noteworthy that the basic chromosome number in *D.saccata* is n = 7, which is shared only with *D.pendula*, *D.hepta* Pala, Casu, & Vacca, 1981, *D.batuensis* Ball, 1970, and *D.ryukyuensis* Kawakatsu, 1976 (Kawakatsu et al. 1976; Pala et al. 1981; Khang et al. 2017; Wang et al. 2024). However, *D.pendula* exhibits an aneuploid plus mixoploid karyotype, with diploid (2n = 2x = 14 + 0-1 B-chromosome) and triploid (2n = 3x = 21 + 0-1 B-chromosome) sets, while *D.batuensis* exhibits six metacentric chromosomes and one subtelocentric chromosome, and *D.ryukyuensis* shows six metacentric chromosomes and one submetacentric chromosome, which is the case also in *D.hepta*. In contrast, *D.saccata* exhibits a mixoploid karyotype with diploid (2n = 2x = 14) and triploid (2n = 3x = 21) sets, with all chromosomes being metacentric, thus contrasting with the chromosome complements of the other species.

In fact, *D.saccata* produced infertile cocoons and only showed asexual reproduction by means of fission, which corresponds with its poorly developed or hyperplasic ovaries and the triploid chromosome complement. It has been established that in such abnormal ovaries the oocytes are anomalous, thus preventing regular oogenesis (Harrath et al. 2014).

##### ﻿ Dugesia aconcinna

Taxon classificationAnimaliaTricladidaDugesiidae

Chen & Dong sp. nov.

35B9C03B-4424-5392-A6F6-9A46B4149A49

https://zoobank.org/1DBF6C63-D76D-46F8-B958-E807694E8639

**Figure 7.**
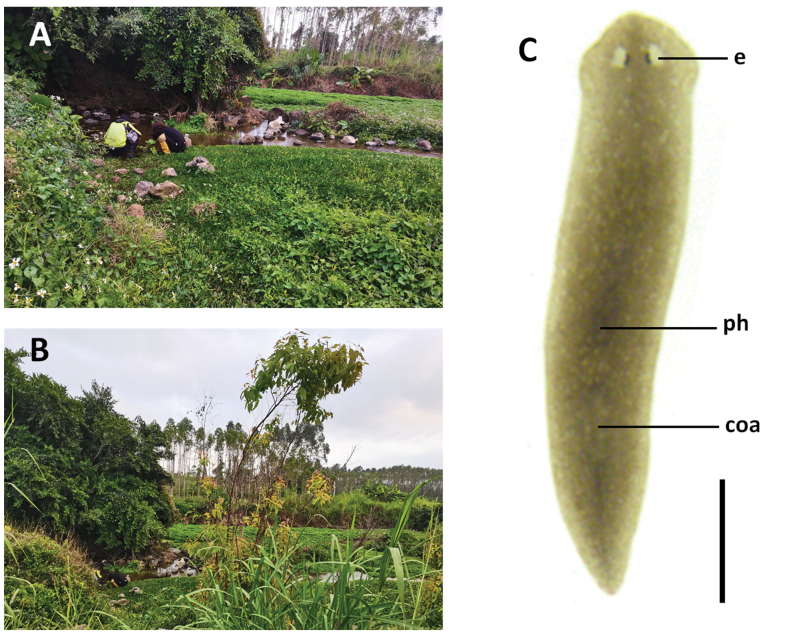
Habitat and external appearance of *Dugesiaaconcinna***A, B** sampling site and habitat **C** sexually mature, live individual. Abbreviations: coa: copulatory apparatus; e: eye; ph: pharynx. Scale bar: 2 mm.

**Figure 8.**
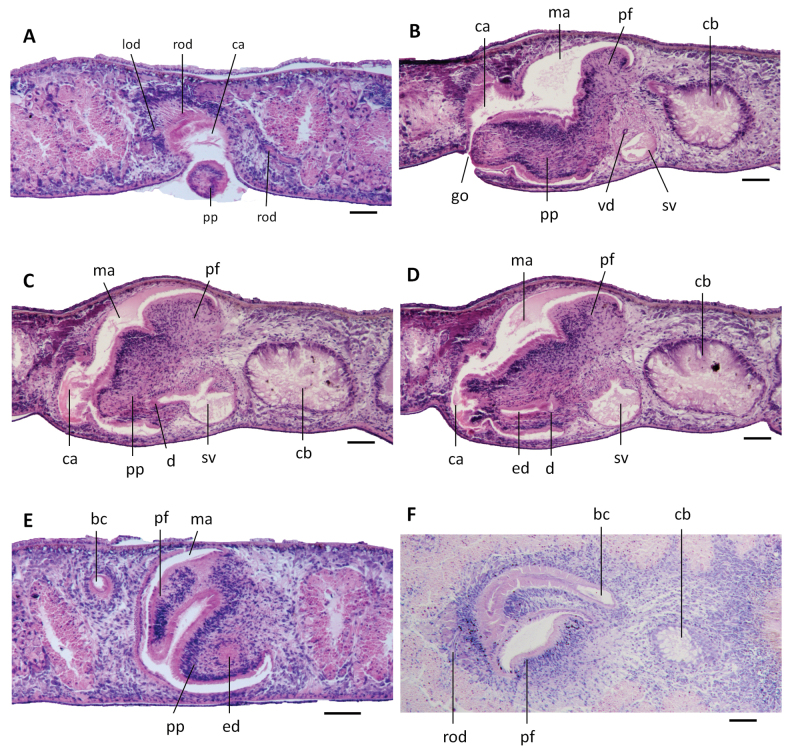
*Dugesiaaconcinna*. Photomicrographs of sagittal **(B–D**), transverse (**A, E**) and horizontal (**F**) sections. **A** Paratype ZMHNU-TPYC8, showing penis papilla, common atrium, left oviduct and right oviduct **B** holotype ZMHNU-TPYC5, showing copulatory bursa, seminal vesicle, vasa deferentia, penis papilla, penis fold, male atrium, common atrium, and genital pore **C** holotype ZMHNU-TPYC5, showing copulatory bursa, seminal vesicle, diaphragm, penis papilla, penial fold, male atrium, and common atrium **D** holotype ZMHNU-TPYC5, showing copulatory bursa, seminal vesicle, diaphragm, ejaculatory duct, penial fold, male atrium, and common atrium **E** paratype ZMHNU-TPYC8, showing penis papilla, ejaculatory duct, penial fold, male atrium, and bursal canal **F** paratype ZMHNU-TPYC9, showing copulatory bursa, bursal canal, and penis fold. Abbreviations: bc: bursal canal; ca: common atrium; cb: copulatory bursa; d: diaphragm; ed: ejaculatory duct; go: gonopore; lod: left oviduct; ma: male atrium; pf: penial fold; pp: penis papilla; rod: right oviduct; sv: seminal vesicle; vd: vas deferens. Scale bars: 100 μm.

**Figure 9.**
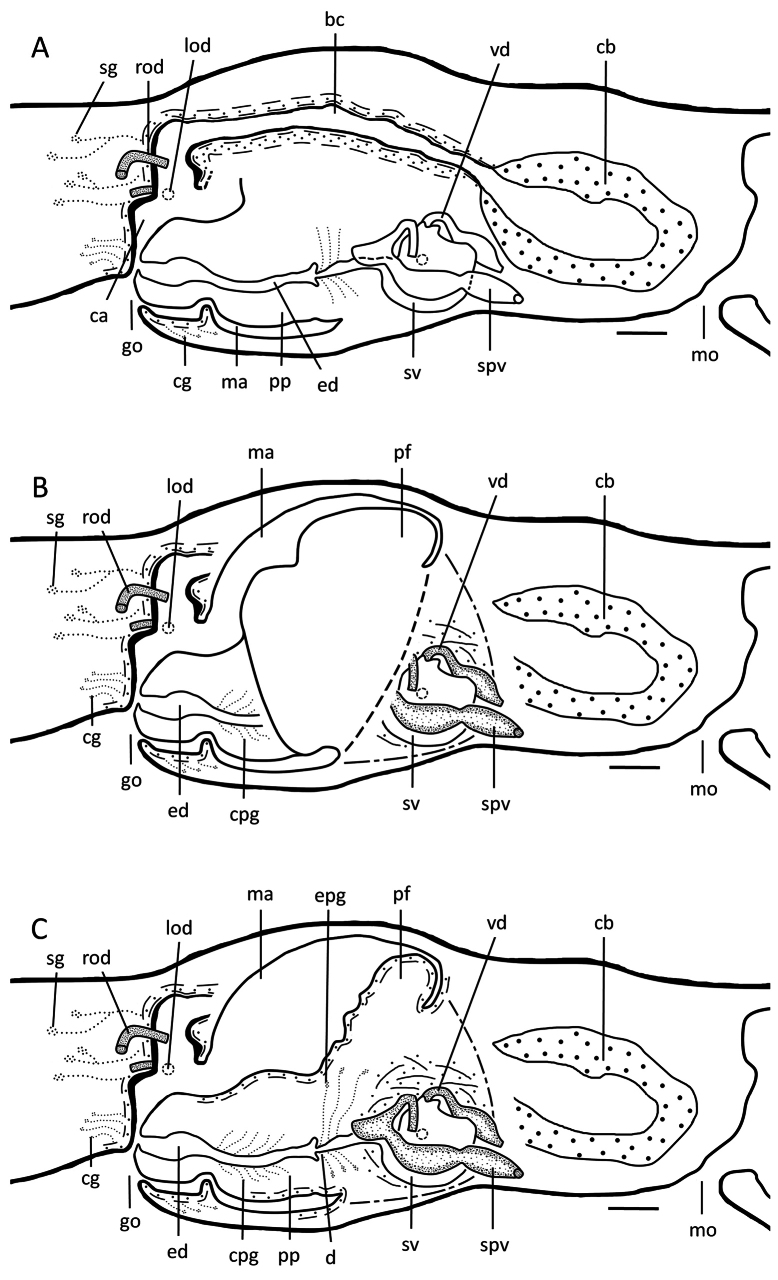
*Dugesiaaconcinna*. Sagittal reconstruction of the copulatory apparatus of holotype TPYC5. **A** Female copulatory apparatus **B** penial fold **C** male copulatory apparatus. Abbreviations: bc: bursal canal; ca: common atrium; cb: copulatory bursa; cg: cement glands; cpg: cyanophil penial glands; d: diaphragm; ed: ejaculatory duct; epg: extrabulbar penial glands; go: gonopore; lod: left oviduct; ma: male atrium; mo: mouth; pf: penial fold; pp: penis papilla; rod: right oviduct; spv: spermiducal vesicles; sg: shell glands; sv: seminal vesicle; vd: vas deferens. Scale bars: 100 μm.

Figs 1 , 2 , 7 , 8 , 9

###### Collection site, habitat, and reproduction.

On 4 January 2019, the specimens were collected from a stream in the Qingfeng village, Leizhou Peninsula (Figs 1, 7A, B), which is a volcanic spring at an altitude of 56 m a.s.l.; air temperature was 22 °C and water temperature was 19 °C. With respect to the *D.aconcinna* population, six mature worms and five asexual worms were collected in the field. After ~ 5 months under laboratory conditions, all of the immature worms sexualised, although none of the worms (sexual in the field and the sexualised ones in the laboratory) produced any cocoons.

###### Material examined.

***Holotype*** • ZMHNU-TPYC5, Qingfeng village (21°14'33"N, 110°9'49"E), alt. 56 m a.s.l., Suixi County, Guangdong Province, China, 4 January 2019, coll. Z-M Dong, L Wang and J-Z Chen, sagittal sections on 14 slides. ***Paratypes*** • RMNH.VER.22250.1, ibid., sagittal sections on 11 slides • RMNH.VER.22250.2, ibid., sagittal sections on 10 slides • ZMHNU-TPYC1-3, 6, 7,11, ibid., sagittal sections on 14, 29, 15, 5, 21, 14 slides • ZMHNU-TPYC9, ibid., horizontal sections on 23 slides • ZMHNU-TPYC8, ibid., transverse sections on 19 slides.

###### Diagnosis.

*Dugesiaaconcinna* is characterised by the presence of the following features: live, mature animals rather small; asymmetrical openings of the oviducts into the common atrium; vasa deferentia separately opening into the posterior portion of the seminal vesicle; penis papilla of a very characteristic shape, with the part housing the connecting duct, diaphragm, and ejaculatory duct being a cylindrical structure with a blunt tip, while at its right-hand side sits a large penial fold that attaches to the base of the penis papilla; ejaculatory duct following a ventrally displaced course through the penis papilla, after which it opens at the tip of the papilla; presence of a duct between diaphragm and seminal vesicle.

###### Description.

Body of both asexual and sexual live specimens is quite small, with the sexual worms being only 6–9 mm in length and 1.0–1.2 mm in width. The low-triangular head is provided with two blunt auricles and two eyes, which are placed in pigment-free spots. Each pigmented eyecup houses numerous photoreceptor cells. The dorsal surface is yellow-brown, with many brown pigment granules and pale blotches all over the dorsal surface; the ventral surface is paler than the dorsal body surface (Fig. 7C).

Pharynx situated in the mid-region of the body, measuring ~ 1/6 of the body length. Mouth opening located at the posterior end of the pharyngeal pocket. Outer pharyngeal musculature is composed of a thin, subepithelial layer of longitudinal muscles, followed by a thin layer of circular muscles; no extra inner layer of longitudinal muscles was observed. The inner pharyngeal musculature consists of a thick, subepithelial layer of circular muscle, followed by a thin layer of longitudinal muscle.

In those specimens in which we were able to examine the ovaries, most of the gonads were not hyperplasic (specimens TPYC3, 5, 7, 8, and RMNH.VER.22250.1), excepting specimens TPYC6, and 11, and RMNH.VER.22250.2. In general, the oval ovaries are situated at 1/3–1/4 of the distance between the brain and the root of the pharynx, occupying ~ 1/4 of the dorso-ventral space. The oviducts arise from the dorsal wall of the ovaries, then turn to the ventral side and run in a caudal direction to the level of the genital pore. Subsequently, the left oviduct bends dorsally to open into the common atrium, while the right oviduct exhibits a much more pronounced curvature towards the dorsal body surface, after which it recurves in antero-ventral direction to open into the most ventral, proximal portion of the bursal canal, at the point where the latter communicates with the common atrium (Figs 8A, 9A). Thus, the right oviducal branch opens dorsally to the left one. The oviducts are lined with a columnar, infranucleated epithelium.

The sac-shaped copulatory bursa lies immediately behind the pharyngeal pocket and may occupy the entire dorso-ventral space or, at least, a considerable portion of it. The bursa is lined with a columnar, vacuolated epithelium with basal nuclei and is devoid of any surrounding musculature (Figs 8B–D, F, 9). Near its communication with the postero-dorsal section of the bursa, the bursal canal is rather narrow and may occupy ~ 1/8 of the dorso-ventral space (Figs 8F, 9A). From thereon, the canal expands somewhat in diameter while it runs in a caudal direction to the left side of the male copulatory apparatus. At the level of the gonopore, the posterior section of the canal exhibits a rather abrupt, ventrally directed bend, after which it opens into the common atrium (Fig. 9A). The bursal canal is lined with cylindrical, infranucleated, ciliated cells and is surrounded by a subepithelial layer of longitudinal muscles, followed by a layer of circular muscle; an extra outer layer of longitudinal musculature, forming the ectal reinforcement, extends from the copulatory bursa to the atrium. Erythrophil shell glands open into the vaginal region of the bursal canal, near the oviducal openings.

The numerous, well-developed testes are situated dorsally and provided with mature spermatozoa. On either side of the midline of the body, testicular follicles are arranged in eight or nine longitudinal zones and extend from the posterior level of the ovaries to almost the posterior end of the body.

At the level of the pharyngeal pocket, the vasa deferentia expand to form spermiducal vesicles, which are packed with mature spermatozoa (Fig. 9). Upon reaching the large penis bulb, the vasa deferentia turn dorso-medially and quickly decrease very much in diameter while penetrating the wall of the bulb. Subsequently, the sperm ducts open separately and symmetrically into the mid-posterior section of the seminal vesicle, near the point where it communicates with the connecting duct that leads to the diaphragm. The sperm ducts are lined with a nucleated epithelium and surrounded by a layer of circular muscle.

The voluminous, oval seminal vesicle is lined by a flat, nucleated epithelium and is surrounded by a layer of intermingled muscle fibres. The seminal vesicle occupies ~ 2/5 of the dorso-ventral space and is located in the ventral portion of the penis bulb, close to the ventral body surface (Figs 8B–D, 9). Although the penis bulb is rather shallow, it is, nevertheless, a large structure, occupying almost the entire dorso-ventral space (Figs 8B–D, 9). A relatively long and broad duct connects the seminal vesicle with a small diaphragm, the latter leading to the ejaculatory duct (Figs 8C, D, 9). This interconnecting duct is lined by an infranucleated epithelium and is surrounded by a layer of intermingled muscle fibres. The small diaphragm is located at the level of the root of the penis papilla and receives the abundant secretion of erythrophil penis glands (Figs 8C, D, 9). Both the connecting duct and the ejaculatory duct run a ventrally displaced course through the penis papilla, with the relatively broad ejaculatory duct opening at the tip of the papilla (Figs 8D, F, 9). The ejaculatory duct is lined with a cuboidal, infranucleated epithelium and is devoid of any surrounding musculature.

The penis papilla has a very characteristic shape. The part that houses the connecting duct, diaphragm, and ejaculatory duct is a cylindrical structure with a blunt tip. This seems to be a rather symmetrical portion of the papilla but it should be noted that it concerns a lateral, left-hand part of the penis papilla. The right-hand part of the papilla develops a large penial fold (Figs 8E, 9B) that attaches to the base of the penis papilla. Penis papilla and penial fold are covered with a nucleated epithelium, which is underlain by a subepithelial layer of circular muscle, followed by a layer of longitudinal muscle fibres (Figs 8B–F, 9).

The genital atrium is divided into a common atrium and male atrium. The common atrium communicates with a gonoduct, which leads to the ventral gonopore; the gonoduct is lined by a columnar epithelium and receives the openings of cement glands (Fig. 9).

###### Etymology.

The specific epithet is derived from the Latin adjective *aconcinna*, asymmetrical, and alludes to the asymmetrical penial fold as well as the asymmetrical oviducal openings into the bursal canal.

###### Discussion.

A good number of *Dugesia* species possesses so-called penial annexes in the form of penial folds, which sometimes were indicated by the term adenodactyl. However, the term adenodactyl should not be applied to these penial annexes (Stocchino et al. 2017). Penial folds are located at the base of the penial papilla and are usually covered by the musculature of the penis bulb; folds may be of the parenchymatic-muscular type or of the parenchymatic-musculo-glandular type (Stocchino et al. 2017). Furthermore, penial folds may be located at both the ventral and dorsal side of the penis papilla, albeit the ventral fold may be smaller than the dorsal one, or a single fold may be restricted to the dorsal, dorso-lateral, or lateral portion of the papilla. Such a single fold is present in ~ 20 species of *Dugesia*. For the present comparative discussion, it suffices to concentrate on those species that exhibit a dorso-lateral or lateral penial fold more or less comparable to that of *D.aconcinna*, viz., *D.arcadia* de Vries, 1988, *D.benazzii* Lepori, 1951, *D.golanica* Bromley & Benazzi, 1991, *D.hoidi* Dols-Serrate, Stocchino & Riutort, 2024, *D.iranica* Livanov, 1951, *D.libanica* Bromley & Benazzi, 1991, *D.mariae* Stocchino, Dols-Serrate & Riutort, 2023, *D.minotauros* de Vries, 1984. However, all of these species differ from *D.aconcinna* in the absence of a connecting duct between the seminal vesicle and the diaphragm, perhaps excepting *D.izuensis* Katô, 1943 (cf. Sluys et al. 1998: table II; Dols-Serrate et al. 2024). For *D.izuensis* a diaphragm was not described but it is presumed that the abundant openings of eosinophilic penial glands approximately halfway into the ejaculatory duct (Kato 1950; Kawakatsu 1983) coincides with the location of the diaphragm, which is presumably very small. However, in other aspects *D.izuensis* is rather different from *D.aconcinna*. For example, in *D.izuensis* the penis papilla is a massive and plump structure, whereas the papilla in *D.aconcinna* is cylindrical. Other differences concern the penial fold. In *D.izuensis* the fold has the shape of a conical papilla with the central part filled with cyanophilic secretion, in contrast to the flap-like fold of *D.aconcinna* that lacks any secretions.

With respect to the shape and position of its penial fold, *D.aconcinna* resembles *D.benazzii*, *D.hoidi*, and *D.mariae*, all of which possess a flap-like penial fold that extends dorso-laterally of the penis papilla, which holds true also for *D.minotauros* (de Vries 1984; Dols-Serrate et al. 2024). However, in all of these species the fold is situated on the left side of the penis papilla, in contrast to *D.aconcinna* in which the fold extends over the right side of the papilla. But there are also other differences. In *D.benazzii* and *D.hoidi*, the two vasa deferentia follow highly asymmetrical trajectories before opening, equally asymmetrically, into the seminal vesicle. In contrast, in both *D.mariae* and *D.aconcinna* the sperm ducts follow symmetrical trajectories. In *D.iranica* the penial fold sits also on the left side of the penis papilla, albeit it is not a flap-like fold, but a conical structure of variable size, which sometimes may be as large as the penis papilla; it is of the musculo-glandular type. All of this is different from the situation in *D.aconcinna*. Similarly to *D.aconcinna*, *D.libanica* possesses also a relatively long, cylindrical penis papilla with a blunt tip, with at its right-hand side a well-developed penial fold. In contrast to *D.aconcinna*, the fold of *D.libanica* is not flap-like but consists of a pear-shaped papilla, located dorsally to the right of the penis papilla, that may reach a length of ~ 3/4 of the penial papilla (Bromley and Benazzii 1991). Notably, none of above-mentioned species belongs to the same clade as *D.aconcinna*. Although, these species (*D.adunca*, *D.ancoraria*, *D.bengalensis*, and *D.notogaea*) are closely related molecularly, they can be easily distinguished from *D.aconcinna* by anatomical features.

## ﻿General discussion

Molecular phylogenetic trees based on the concatenated dataset showed a basically identical topology with previous studies (Stocchino et al. 2017; Wang et al. 2021a, 2022; Chen et al. 2022). The clade comprising the Malagasy and Afrotropical/Mediterranean taxa is sister to the other major lineage in the phylogenetic tree of the genus *Dugesia*, comprising representatives from all other regions of the world. The Western Palearctic group shares a sister-group relationship with the Eastern Palearctic/Oriental/Australasian group. However, for *Dugesia*, the available molecular data and markers are limited, and the known species of China are scarce. In turn, this causes several nodes to have low support values in BI and ML trees (Fig. 1, Suppl. material 3).

Previous studies showed that the lowest *COI* and *ITS-1* distance values between species are usually higher than 6% and 1%, respectively (Lázaro et al. 2009; Solà et al. 2013). *Dugesiasaccata* showed the lowest *COI* and *ITS-1* distances with *D.deharvengi* and *D.pendula*, being 8.78% and 1.76%, respectively. *Dugesiaaconcinna* showed the lowest *COI* and *ITS-1* distances with *D.deharvengi and D.adunca*, being 10.87% and 2.97%, respectively. Therefore, *D.aconcinna* and *D.saccata* are well-separated from their congeners, which further supports their separate specific status as suggested by the morphological and phylogenetic analyses.

It is noteworthy that although *D.saccata*, *D.semiglobosa*, and *D.majuscula* are from Hainan Island, *D.saccata* shares only a distant relationship to *D.semiglobosa*. Furthermore, *D.saccata* differs anatomically greatly from *D.semiglobosa*, in that *D.saccata* has a duct between the seminal vesicle and the diaphragm, whereas *D.semiglobosa* has two diaphragms without a duct (Wang et al. 2021b). In contrast to *D.semiglobosa*, *D.saccata* exhibits a rather close relationship with *D.majuscula*, but the ejaculatory duct in *D.majuscula* has a dorsal opening at the tip of the papilla, contrasting with the subterminal and ventral opening in *D.saccata* (Wang et al. 2021b). Our results suggest that different *Dugesia* lineages were already present in the Hainan area prior to its isolation as an island.

## Supplementary Material

XML Treatment for Dugesia saccata

XML Treatment for Dugesia aconcinna

## ﻿Additional information

### Author ORCIDs

Lei Wang https://orcid.org/0000-0003-2328-1530

Yi-Fang Chang https://orcid.org/0009-0007-1018-8392

Xin-Xin Sun https://orcid.org/0000-0001-8167-1495

Ronald Sluys https://orcid.org/0000-0002-9776-3471

De-Zeng Liu https://orcid.org/0000-0002-9354-2073

Zi-Mei Dong https://orcid.org/0000-0002-6416-590X

Guang-Wen Chen https://orcid.org/0000-0001-8723-523X

### Data availability

All of the data that support the findings of this study are available in the main text or Supplementary Information. Holotypes and paratypes of the two new species were deposited in the Zoological Museum of the College of Life Science of Henan Normal University, Xinxiang, China (ZMHNU), and Naturalis Biodiversity Center, Leiden, The Netherlands (RMNH).

## Supplementary materials

Supplementary material 1 Primer sequences used for PCR amplification Data type docx https://binary.pensoft.net/file/1296563This dataset is made available under the Open Database License (http://opendatacommons.org/licenses/odbl/1.0/). The Open Database License (ODbL) is a license agreement intended to allow users to freely share, modify, and use this Dataset while maintaining this same freedom for others, provided that the original source and author(s) are credited.Lei Wang, Yi-Fang Chang, Xin-Xin Sun, Ronald Sluys, De-Zeng Liu, Zi-Mei Dong, Guang-Wen Chen

Supplementary material 2 Extended methods Data type docx https://binary.pensoft.net/file/1296564This dataset is made available under the Open Database License (http://opendatacommons.org/licenses/odbl/1.0/). The Open Database License (ODbL) is a license agreement intended to allow users to freely share, modify, and use this Dataset while maintaining this same freedom for others, provided that the original source and author(s) are credited.Lei Wang, Yi-Fang Chang, Xin-Xin Sun, Ronald Sluys, De-Zeng Liu, Zi-Mei Dong, Guang-Wen Chen

Supplementary material 3 Molecular phylogenetic tree obtained from ML analysis of the concatenated dataset Data type tif Explanation note Numbers at nodes indicate support values (pp). New species indicated in red. Scale bar: substitutions per site. https://binary.pensoft.net/file/1296565This dataset is made available under the Open Database License (http://opendatacommons.org/licenses/odbl/1.0/). The Open Database License (ODbL) is a license agreement intended to allow users to freely share, modify, and use this Dataset while maintaining this same freedom for others, provided that the original source and author(s) are credited.Lei Wang, Yi-Fang Chang, Xin-Xin Sun, Ronald Sluys, De-Zeng Liu, Zi-Mei Dong, Guang-Wen Chen

Supplementary material 4 Genetic distances for *COI* Data type xls Explanation note Highest and lowest distance values between the two new species and congeners indicated in blue and red, respectively. Green: distance value between the two new species. https://binary.pensoft.net/file/1296566This dataset is made available under the Open Database License (http://opendatacommons.org/licenses/odbl/1.0/). The Open Database License (ODbL) is a license agreement intended to allow users to freely share, modify, and use this Dataset while maintaining this same freedom for others, provided that the original source and author(s) are credited.Lei Wang, Yi-Fang Chang, Xin-Xin Sun, Ronald Sluys, De-Zeng Liu, Zi-Mei Dong, Guang-Wen Chen

Supplementary material 5 Genetic distances for *ITS-1* Data type xls Explanation note Highest and lowest distance values between the two new species and congeners indicated in blue and red, respectively. Green: distance value between the two new species. https://binary.pensoft.net/file/1296567This dataset is made available under the Open Database License (http://opendatacommons.org/licenses/odbl/1.0/). The Open Database License (ODbL) is a license agreement intended to allow users to freely share, modify, and use this Dataset while maintaining this same freedom for others, provided that the original source and author(s) are credited.Lei Wang, Yi-Fang Chang, Xin-Xin Sun, Ronald Sluys, De-Zeng Liu, Zi-Mei Dong, Guang-Wen Chen
