# Supplementary material for: ﻿Two new species of freshwater planarian from Hainan Island and Leizhou Peninsula, southern China (Platyhelminthes, Tricladida, Dugesiidae)
Source: Zookeys. 2025 Apr 1;1233:289–313. doi: 10.3897/zookeys.1233.142976 (PMC11979614; doi:10.3897/zookeys.1233.142976)
Supplement: Supplementary material 1 — Primer sequences used for PCR amplification [file zookeys-1233-289_article-142976__-s001.docx]

**Two new species of freshwater planarian from** **Hainan Island and** **Leizhou Peninsula, southern China (Platyhelminthes, Tricladida, Dugesiidae, *Dugesia*)**

Lei Wang^1^, Yi-Fang Chang^1^, Xin-Xin Sun^1^, Ronald Sluys^2^, De-Zeng Liu^1^, Zi-Mei Dong^1*^, Guang-Wen Chen^1*^

**^1^***College of Life Science, Henan Normal University, Xinxiang, 453007 Henan, China*

**^2^***Naturalis Biodiversity Center, Leiden, The Netherlands*

^*^ Corresponding author: Guang-Wen Chen (chengw0183@sina.com), Zi-Mei Dong (dzmhjx@163.com)

**Table S1** Primer sequences used for PCR ampliffcation.

| Gene | Primer | Sequence (5’-3’) | Reference | PCR protocol |
| --- | --- | --- | --- | --- |
| *18S rDNA* | 18S 1F | Forward: TACCTGGTTGATCCTGCCAGTAG | Carranza et al., 1996 | 5 min 94 °C, 40×(50 s 95 °C, 45s 48 °C, 50 s 72 °C), 7 min 72 °C. |
|  | 18S 9R | Reverse: GATCCTTCCGCAGGTTCACCTAC |  |  |
| *28S rDNA* | 28S MF1F | Forward: GTTGTGTTTTTAATTGAACAG | Stocchino et al., 2017 | 5 min 94 °C, 40×(50 s 95 °C, 45s 43 °C, 50 s 72 °C), 7 min 72 °C. |
|  | 28S MR1F | Reverse: TGCAGACTTTAGATC |  |  |
|  | 28S MF2R | Forward: TCTTAATATGYGGTTG |  |  |
|  | 28S MR2R | Reverse: CTCCACTCTGACTTAC |  |  |
| *ITS-1* | ITS9F | Forward: GTA GGT GAA CCT GCG GAA GG | Baguñà et al. 1999 | 5 min 94 °C, 30×(30s 98 °C, 45s 46 °C, 30s 72 °C), 7 min 72 °C. |
|  | ITSR | Reverse: TGC GTT CAA ATT GTC AAT GAT C |  |  |
| *COI* | BarTF | Forward: ATAGGTGGKTTTGGTAAT | Lázaro et al., 2009 | 5 min 94 °C, 35×(50 s 94 °C, 45 s 50 °C, 45 s 68 °C), 7 min 72 °C. |
|  | COIR | Reverse: ATTWAYAWCAACACTACGAC |  |  |
