# Supplementary material for: ﻿Two new species of freshwater planarian from Hainan Island and Leizhou Peninsula, southern China (Platyhelminthes, Tricladida, Dugesiidae)
Source: Zookeys. 2025 Apr 1;1233:289–313. doi: 10.3897/zookeys.1233.142976 (PMC11979614; doi:10.3897/zookeys.1233.142976)
Supplement: Supplementary material 2 — Extended methods [file zookeys-1233-289_article-142976__-s002.docx]

**Two new species of freshwater planarian from** **Hainan Island and** **Leizhou Peninsula, southernChina (Platyhelminthes, Tricladida, Dugesiidae, *Dugesia*)**

Lei Wang^1^, Yi-fang Chang^1^, Xin-Xin Sun^1^, Ronald Sluys^2^, De-Zeng Liu^1^, Zi-Mei Dong^1*^, Guang-Wen Chen^1*^

**^1^***College of Life Science, Henan Normal University, Xinxiang, 453007 Henan, China*

**^2^***Naturalis Biodiversity Center, Leiden, The Netherlands*

^*^ Corresponding author: Guang-Wen Chen (chengw0183@sina.com), Zi-Mei Dong (dzmhjx@163.com)

**Extended Methods Karyology**

5-10 individuals were randomly selected, with each of the worms being treated separately. Regenerating blastemas were obtained by transversely cutting each worm into 3 fragments (head, middle, tail), which were cultured for 3 days. After 3 days, 4 blastemas (head, prepharyngeal, post-pharyngeal, tail) were cut off transversely and placed on 4 separate slides and, thereafter, treated with a 0.02% colchicine solution at 4 °C for 3 h. Hereafter, the blastemas were placed in 0.1% KCl hypotonic solution for 3 h. Subsequently, the surplus hypotonic solution was removed from the slides, and then fixative fluid I (glacial acetic acid: absolute alcohol: deionized water in the ratios 3:3:4) was added and was removed after only a few seconds. Fixative fluid I was then added again for about 30 s, after which the blastemas were evenly hammered with an oversized needle in order to create a cell suspension, which spread over the glass slide. Before the slides were completely dry, fixative fluid II (glacial acetic acid: absolute alcohol as in 1:1 proportion) was drop-wise added to the preparations. When the slides had dried again, fixative fluid III (glacial acetic acid) was added for only a few seconds, after which it was removed and the preparations were dried at room temperature. After 24 h, they were stained for 15 min. with a 0.5% Giemsa solution, whereafter the slides were washed with deionized water and dried at room temperature. Hereafter, coverslips were attached with DPX mountant (MFD1266, MesGen Biotechnology Co.,Ltd., Shanghai, China).

Mitotic metaphase chromosomes were observed and photographed under a compound microscope (ZEISS, Axio Scope. A1) equipped with CoolCube digital camera (MetaSystems, Altlussheim, Germany). Karyograms were prepared using the IKAROS Karyotyping system (MetaSystems, Altlussheim, Germany, https://metasystems-international.com/en/products/ikaros/). Well-spread sets of metaphase plates from randomly selected individuals were used to determine ploidy level, centromeric indices, and relative lengths of the chromosomes. Based on IKAROS artificial intelligence (AI) for karyotyping, the curved or overlapping chromosomes were accurately measured and their centromeric position calculated. Hereafter, relative lengths of the chromosomes and centromeric positions were re-checked by manual calibration. In the calculations of the averages, the very few heteromorphic chromosomes and karyotypes were not included, in order to avoid misleading results and incorrect conclusions. Karyotype parameter measurements were executed as published previously by Chen et al. (2008). Chromosomal nomenclature followed Levan et al. (1964).

**References**

Chen G-W, Wang Y-L, Wang H-K, Fu R-M, Zhang J-F, Liu D-Z (2008) Chromosome and karyotype analysis of *Polycelis wutaishanica* (Turbellaria, Tricladida) from Shanxi province, China. Acta Zootaxonomica Sinica 33(3): 449–452.

Levan A, Fredga K, Sandberg AA (1964) Nomenclature for centromeric position on chromosomes. Hereditas 52: 201–220. https://doi.org/10.1111/j.1601-5223.1964.tb01953.x
