# Supplementary figures and images for: ﻿Two new species of freshwater planarian from Hainan Island and Leizhou Peninsula, southern China (Platyhelminthes, Tricladida, Dugesiidae)
Source: Zookeys. 2025 Apr 1;1233:289–313. doi: 10.3897/zookeys.1233.142976 (PMC11979614; doi:10.3897/zookeys.1233.142976)

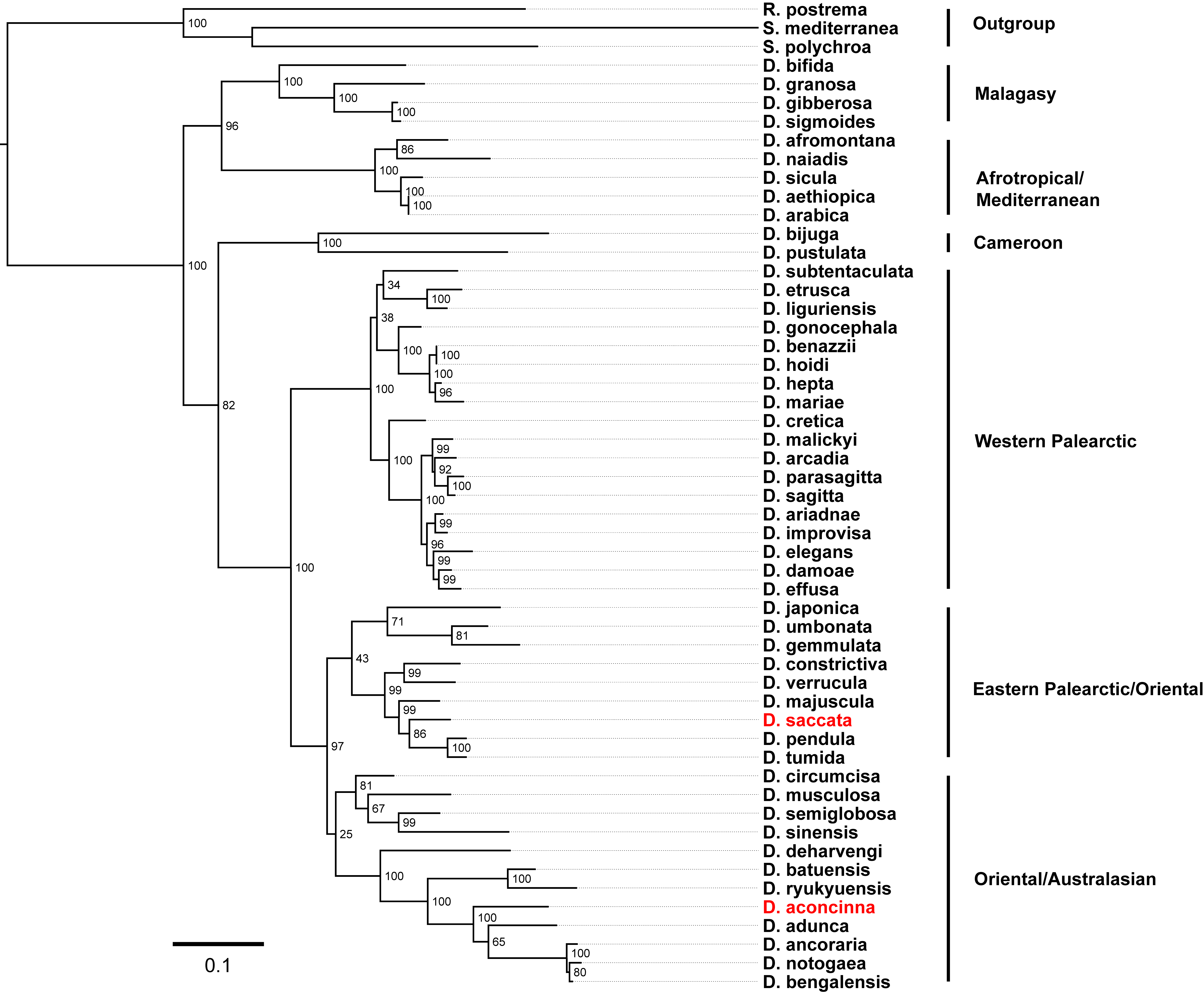

Supplement: Supplementary material 3 — Molecular phylogenetic tree obtained from ML analysis of the concatenated dataset [file zookeys-1233-289_article-142976__-s003.tif]
